# Analysing the implementation of infection prevention and control measures in health care facilities during the COVID-19 pandemic in the African Region

**DOI:** 10.1186/s12879-023-08830-8

**Published:** 2023-11-23

**Authors:** Landry Kabego, Thierno Balde, Deborah Barasa, Babacar Ndoye, Okou-Bisso Hilde, Tendai Makamure, Guy Ohirweoluhya Mulumeoderwa, Trevor Kanyowa, Rashidatu Fouad Kamara, Boiro Hamadou, Opeayo Ogundiran, Joseph Okeibunor, George Williams, Jayne Byakika Tusiime, Phionah Lynn Atuhebwe, Boniface Oyugi, Elande-Taty Mawanda, Andry Razakamanantsoa, Fiona Braka, Dick Chamla, Abdou Salam Gueye

**Affiliations:** World Health Organization/Regional Office for Africa, Brazzaville, Republic of the Congo

**Keywords:** Health care facilities, Health workers, Infection prevention and control, Health care- associated infections

## Abstract

**Background:**

The declaration of SARS-CoV-2 as a public health emergency of international concern in January 2020 prompted the need to strengthen infection prevention and control (IPC) capacities within health care facilities (HCF). IPC guidelines, with standard and transmission-based precautions to be put in place to prevent the spread of SARS-CoV-2 at these HCFs were developed. Based on these IPC guidelines, a rapid assessment scorecard tool, with 14 components, to enhance assessment and improvement of IPC measures at HCFs was developed. This study assessed the level of implementation of the IPC measures in HCFs across the African Region during the COVID-19 pandemic.

**Method:**

An observational study was conducted from April 2020 to November 2022 in 17 countries in the African Region to monitor the progress made in implementing IPC standard and transmission-based precautions in primary-, secondary- and tertiary-level HCFs. A total of 5168 primary, secondary and tertiary HCFs were assessed. The HCFs were assessed and scored each component of the tool. Statistical analyses were done using R (version 4.2.0).

**Results:**

A total of 11 564 assessments were conducted in 5153 HCFs, giving an average of 2.2 assessments per HCF. The baseline median score for the facility assessments was 60.2%. Tertiary HCFs and those dedicated to COVID-19 patients had the highest IPC scores. Tertiary-level HCFs had a median score of 70%, secondary-level HCFs 62.3% and primary-level HCFs 56.8%. HCFs dedicated to COVID-19 patients had the highest scores, with a median of 68.2%, followed by the mixed facilities that attended to both COVID-19 and non-COVID-19 patients, with 64.84%. On the components, there was a strong correlation between high IPC assessment scores and the presence of IPC focal points in HCFs, the availability of IPC guidelines in HCFs and HCFs that had all their health workers trained in basic IPC.

**Conclusion:**

In conclusion, a functional IPC programme with a dedicated focal person is a prerequisite for implementing improved IPC measures at the HCF level. In the absence of an epidemic, the general IPC standards in HCFs are low, as evidenced by the low scores in the non-COVID-19 treatment centres.

**Supplementary Information:**

The online version contains supplementary material available at 10.1186/s12879-023-08830-8.

## Introduction

The SARS-CoV-2 is a positive-sense single-stranded RNA virus that causes severe respiratory infections in humans. SARS-CoV-2, initially detected in Wuhan, China, spread across borders more rapidly to all continents than the previous SARS-CoV. This prompted the World Health Organization (WHO) to declare it a public health emergency of international concern (PHEIC) on 30 January 2020 [1–4].

Globally, on 16 November 2022, WHO reported a cumulative total of 632 953 782 confirmed cases of COVID-19, including 6 593 715 deaths; and in Africa, 9 379 374 confirmed cases, including 175,031 deaths. In the African Region, 1.9% of the confirmed cases were among health workers (HW) [5].

Health workers caring for patients in health care facilities experienced a heavy COVID-19 burden and were disproportionately affected. The prevalence of SARS-CoV-2 infection among them was high, with most of them being asymptomatic carriers [6]. Preventive measures and other conventional procedures were needed to prevent the SARS-CoV-2 virus from passively being transmitted in both the clinical and community settings [7]. To mitigate the spread of infection in the general population, personal protective and social measures, such as handwashing, mask wearing, and physical distancing were introduced [8, 9].

WHO published guidelines for infection prevention and control (IPC) during health care, when COVID-19 was suspected or confirmed. These guidelines emphasize the importance of implementing standard and transmission-based precautions at HCFs, to break the chain of transmission and suppress any further spread of COVID-19. Standard precautions include hand hygiene, respiratory hygiene, use of personal protective equipment, environmental cleaning and disinfection and waste management. Transmission-based precautions for COVID-19 include contact and droplet precautions as well as airborne precautions during aerosol generating procedures [10]. This culminated in WHO developing a rapid assessment scorecard to support Member States in routinely assessing and improving IPC measures in HCFs in the context of COVID-19.

The aim of this study is to determine the status of implementation of IPC measures in HCFs in Africa in the context of the COVID-19 pandemic.

## Methods

### Study design settings and participants

This observational study was conducted in 17 countries in Africa, to assess the implementation status of IPC measures in HCFs, as a strategy to mitigate nosocomial transmission of COVID-19. It was conducted from April 2020 to November 2022, and a total of 5168 HCFs were included in the study. HCFs were strategically chosen based on their location within the hotspots areas, where there was a noticeable rise in Covid-19 cases. This allowed to concentrate this research efforts on areas of potentially higher transmission and, therefore, higher importance for IPC measures.

The HCFs were divided into three categories: primary, secondary and tertiary. They were also categorized according to the type of COVID-19 treatment provided: those dedicated exclusively to COVID-19 patients, those dedicated exclusively to non-COVID-19 patients, and mixed HCFs, where both COVID-19 and non-COVID-19 patients were treated.

### Outcome: IPC compliance measuring

To determine the IPC scores in HCFs, an IPC scorecard rapid assessment tool was developed by the WHO Regional Office for Africa. Upon approval and validation of the tool by the Ministry of Health, the trained IPC focal points conducted HCF assessments and data collection. The tool focuses on 14 priority components (parameters): (i) existence of an IPC programme at the HCF; (ii) triage station; (iii) isolation facility; (iv) hand-wash stations at all the points of care; (v) personal protective equipment (PPE); (vi) waste segregation; (vii) waste disposal; (viii) HW training in basic IPC; (ix) intra-hospital surveillance of COVID-19; (x) sterilization; (xi) cleaning and disinfection of patient environment; (xii) risk assessment of HWs exposed to COVID-19 patients; (xiii) water supply and storage in the HCFs; and (xiv) sanitation and hygiene in the HCFs. Each of the 14 components has three criteria which are the processes, practices and materials or supplies. These 14 priority components used in this tool were determined based on a combination of WHO guidelines, existing literature, and expert consultations. These parameters represent critical aspects of IPC that significantly impact the prevention of nosocomial transmission of Covid-19.

When a component met all the three criteria, it scored 3, when it met two, it scored 2; when it met one, it scored 1, and when it did not meet any criterion, it had a score of 0. The total score was divided by 42 and multiplied by 100 to get the final score in percentage of IPC compliance of each facility. Supplementary material 1 provides the details of the IPC assessment tool.

The IPC assessment tool was digitalized using a Kobo collect form, which was then accessed by the IPC focal points using their mobile phones. This ensured real-time data entry and reduced the potential for manual data entry errors.

To ensure the quality of the survey, several measures were undertaken. Firstly, the IPC rapid facility scorecard assessment tool used in the survey was developed by the renowned WHO experts. This ensures the tool’s credibility and relevance. Furthermore, the assessments and data collection were conducted by IPC focal points who underwent rigorous training specific to the use of this tool. Regular monitoring and quality checks were performed by the national IPC team to ensure the integrity of the collected data.

### Statistical methods

In this study, the R (version 4.2.0) was used for all statistical analyses. A Shapiro–Wilk test for normality was undertaken for numerical data, and the results displayed a non-Gaussian distribution. Numerical data were analysed using the median and interquartile range (IQR). Categorical data were summarized using frequencies or percentages.

The Kruskal–Wallis H test was used to determine if there was a statistically significant difference in IPC scores between the different types of HCFs as well as when comparing the scores between various levels of HCFs in the health system. The Mann–Whitney U test was used to compare the median IPC score between two independent groups, while the Wilcoxon signed-rank test was used to compare the medians of two paired groups. We used the Wilcoxon signed-rank test for the before and after comparison of two follow-up scores.

The difference between groups was regarded as statistically significant when the *P*-value was less than 0.001. A multiple linear regression model was built to analyse independent associations between the level of HCFs, the type of HCFs, the presence or not of an IPC focal point in HCFs, the availability of IPC guidelines in HCFs, training of HWs and trends in IPC scores. This model included variables with a cut-off value of *P* < 0.1 in bivariate analyses. The residual standard error, the adjusted R-squared, F-statistic and its *p*-value were used to evaluate the goodness of fit of the model.

## Results

### Baseline characteristics of the study population

During the study period, 5153 HCFs were assessed; 287 of them were exclusively dedicated to COVID-19 patients, 3791 were dedicated to non-COVID-19 patients and 1075 for both COVID-19 and non-COVID-19 patients (mixed facilities). Primary HCFs numbered 3577, secondary HCFs, 1317, and tertiary HCFs, 259. In total, 11 564 assessments were conducted, which makes an average of 2.2 assessments per HCF.

### Summary of IPC scores for the baseline assessments

For the baseline assessments, a total of 1121 HCFs (21.75%) scored above 79%, 2426 HCFs (47.27%) scored between 50 and 79% and 1596 HCFs (30.97%) scored below 50%. The median IPC score was 60.2% (IQR = 42.9%-78.6%).

**Fig. 1:**
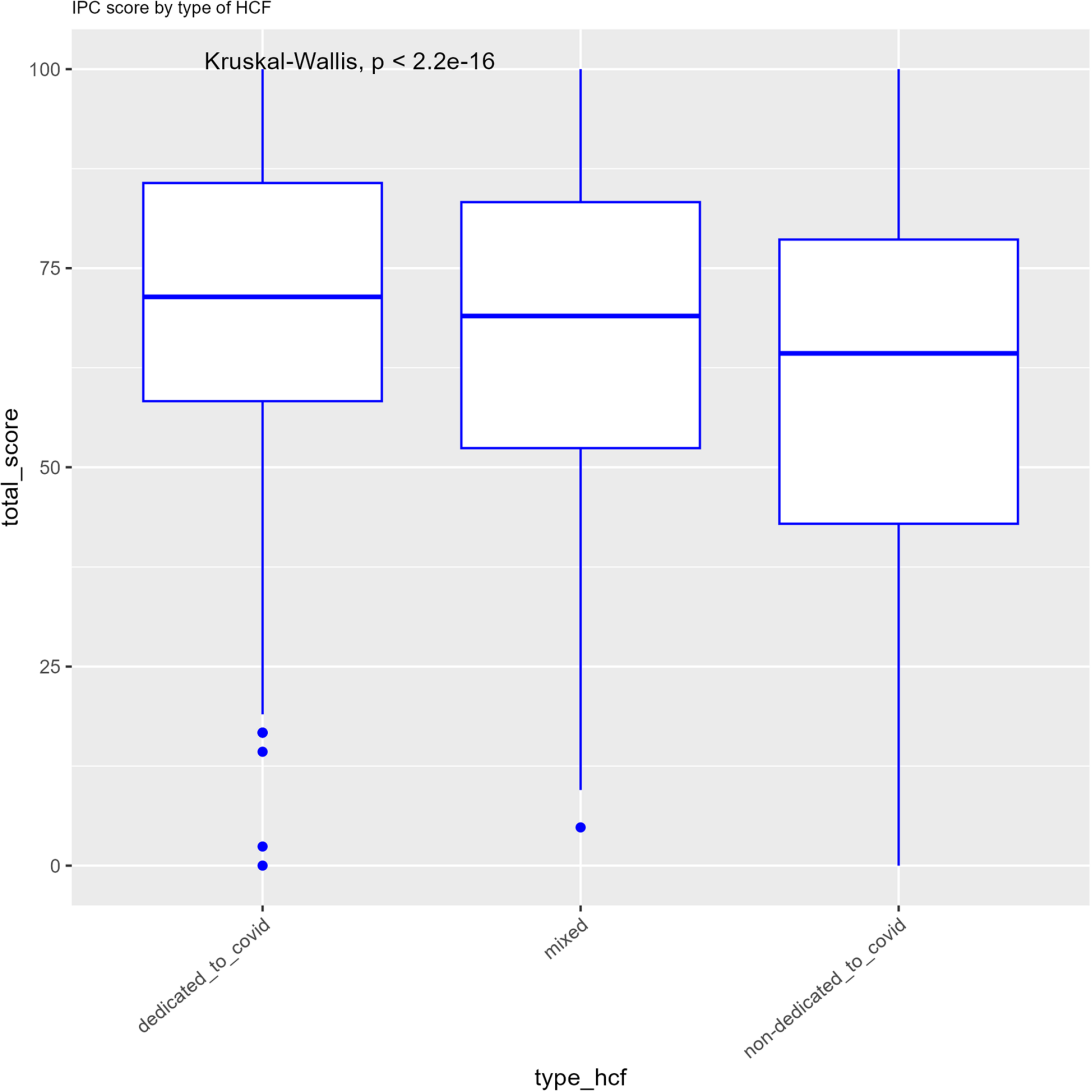
Median IPC scores of health care facilities according to the type of care provided

HCFs dedicated to COVID-19 patients had the highest score [median = 68.2 (IQR = 57.7%-83.3%),] followed by mixed facilities [median = 64.84 (IQR = 50%-81%)] and those dedicated to non-COVID-19 patients [median = 58.4 (IQR = 40.5%-76.2%)] (*p* < 0.001) (see Fig. 1).

**Fig. 2:**
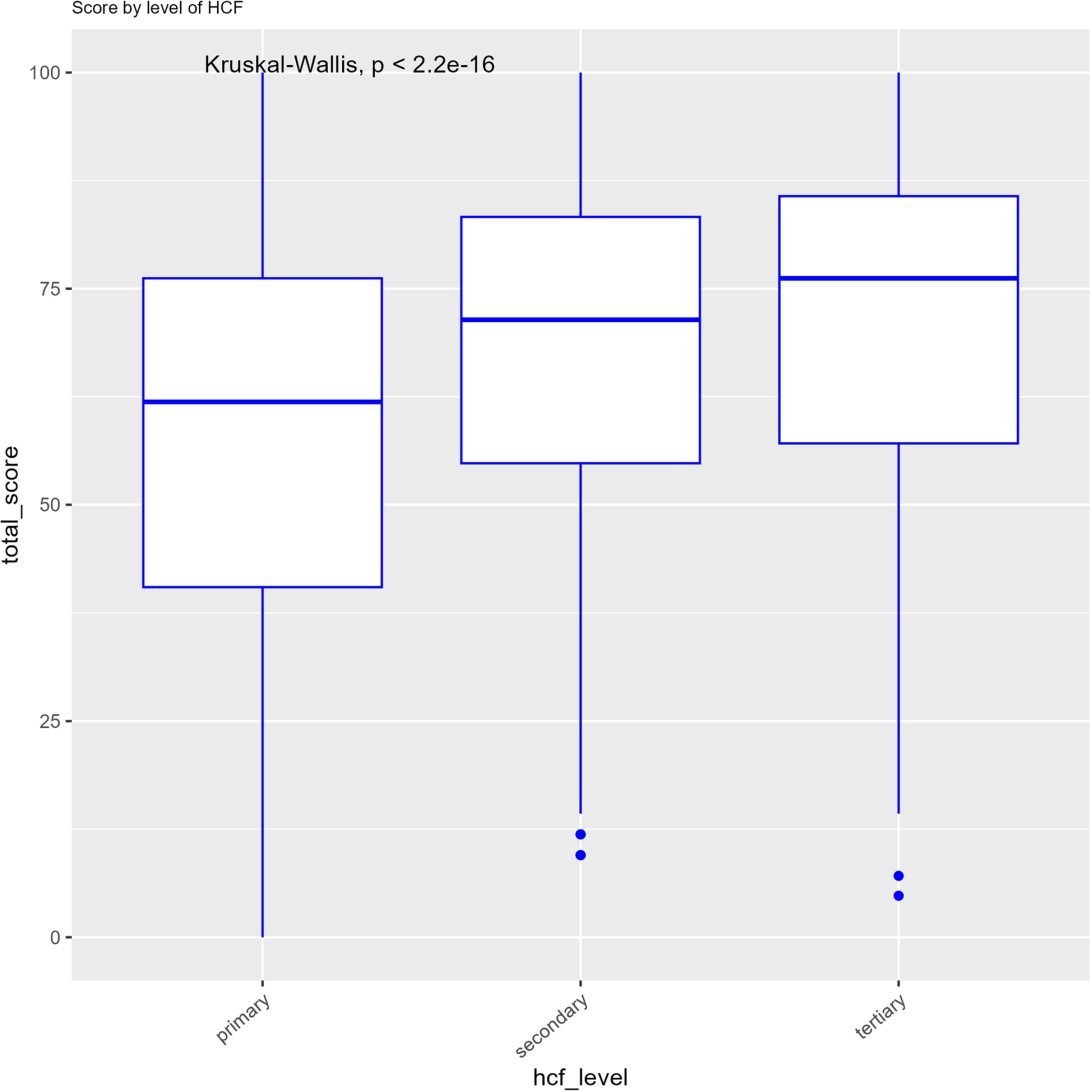
Median IPC scores by level of health care facilities in the health system

Regarding the level of HCFs in the health system, tertiary HCFs had the highest scores with a median score of 70% (IQR = 57.1%-85.7%), followed by secondary HCFs with a median of 62.3%(IQR = 54.8%-81%) and primary HCFs with a median score of 56.8% (IQR = 40.5–73.8) (*p* < 0.001) (see Fig. 2).

### IPC scores variability by IPC components assessed

Only 24.78% of all HCFs met all three criteria for the components, one of which is IPC programmes; 30.25% of HCFs met all the requirements for screening at the entrance of the hospital—24% for hand hygiene and 41% for personal protective equipment. Forty-six per cent of HCFs met all the requirements for waste segregation, 44% for waste disposal and 26% for HW training on IPC.

Table 1 provides the summary of numbers and percentages of HCFs that met the full criteria for each IPC component assessed.

**Table 1:** Summary of the number of health care facilities and percentages of those that meet full requirements per IPC components

IPC components	(*N* = 5163)	Percentage (%)
IPC program	1277	24,78
Triage	1559	30,25
Isolation area	1258	24,41
Hands hygiene	2116	41,06
Personal protective equipment	1274	24,72
Waste segregation	2395	46,48
Waste disposal	2280	44,25
Training on IPC	1389	26,96
Intrahospital surveillance	2047	39,72
Sterilization of reusable materials	1977	38,37
Biocleaning	1867	36,23
Health workers' exposure risk assessment	1840	35,71
Water supply	2209	42,87
Sanitation and hygiene	2918	56,63

IPC scores of HCFs that had appointed IPC focal points were higher [median = 68% (IQR:57.1–81%)] than for HFCs without IPC focal points [median = 41% (IQR = 28.6%-54.8%)], showing a statistically significant (*p* < 0.001) difference.

HCFs with IPC guidelines had a higher IPC median score [median = 70% (IQR = 59.5%-83.3%)] than those without [median = 46.9% (IQR = 33.3%-61.9%)] (*p* < 0.001).

At HCFs where all the HWs had undergone basic IPC training had higher IPC scores [median = 70% (IQR = 59.3%-83.3%)], compared to those where some HWs had not received basic IPC training [median = 48.9% (IQR = 33.3%-64.3%)] (*p* < 0.001).

A multiple linear regression computed showed an association between higher IPC scores with the presence of an IPC focal point in HCFs, the availability of IPC guidelines in HCFs, HCFs that had all their HWs trained in basic IPC, tertiary and secondary HCFs compared to primary HCFs, and dedicated COVID-19 HCFs compared to non-dedicated ones. This model found *p* < 0.001 for all the listed predicators.

The fitted regression model was: IPC score = 36.89–1 0.76 (mixed HCF) – 93 (non-dedicated COVID-19) + 6.35(secondary HCF) + 5.13 (tertiary HCF) + 14.67 (Staff trained in IPC) + 12.26 (IPC guidelines in HCFs) + 15.52 (Presence of IPC focal point).

The overall regression was statistically significant (aR^2^ = 0.54, F = 878.4, *p* =  < 0.001). Table 2 provides further information on the model.

**Table 2:** Linear regression predicting IPC scores

Variable	Coefficient	Std Error	T value	Probability
(intercept)	36.89	0.94	38.85	< 0.001
Type of HCF				
Mixed HCF	-1.76	0.97	-1.81	0.07
Non-dedicated Covid-19	-4.93	0.89	-5.54	< 0.001
Level off HCFs				
Secondary	6.35	0.47	13.25	< 0.001
Tertiary	5.13	0.93	5.47	< 0.001
Staff trained in IPC (yes)	14.67	0.42	34.25	< 0.001
IPC guidelines in HCF (yes)	12.26	0.48	25.09	< 0.001
Presence of IPC focal point (yes)	15.52	0.52	29.59	< 0.001
Multiple R-squared	0.54			
Residual standard error	14.65			
Adjusted multiple R-squared	0.54			
F-statistic	878.4			
Prob F-statistic	< 0.001			

### Improvement in IPC scores from the baseline to the follow-up assessments

Among the 5153 HCFs included in this study, 1425 received follow-up assessment. The IPC score was higher for the last assessment [median = 71.4%(IQR = 50%-78%)] (*p* < 0.001) than the first assessment [median score = 64.3% (IQR = 0%-78%)].

## Discussion

This study assessed IPC measures at facility level to mitigate the spread of COVID-19 nosocomial infection in health care settings during the COVID-19 pandemic [10]. Following the baseline HCF assessments, the IPC median score was 60.2%. However, more than 30% of the HCFs presented an average score of lower than 50%. There was an improvement from 64.3% to 71.4% on reassessment of the HCFs that conducted a follow up assessment, highlighting the critical need for monitoring and feedback to improve IPC capacity. In this study, the components on sanitation and hygiene scored the highest, at 56%, while IPC programmes at facility level, presence of an isolation/waiting area, availability and use of PPE and training/education all had scores of less than 30%. Scores of less than 40% were observed in triage, intra-hospital surveillance, sterilization, bio-cleaning and HW risk assessment. While the COVID-19 pandemic was perceived as a stimulus to improve IPC programmes, the implementation of the WHO minimum requirements during the pandemic is noted to be alarmingly low in the African Region. The study highlights and further strengthens evidence on the low IPC capacity at HCF level within the African Region and the need to strengthen IPC programmes through strategic and operational interventions aligned to the WHO minimum requirements [11]. Improving IPC capacity within the African Region will bolster response capacities to public health emergencies, further impact on the quality of patient care, universal health coverage, anti-microbial resistance programmes, and maternal, newborn and child health and other programmes, while simultaneously improving health systems capacity and resilience [12, 13].

Higher scores were noted in facilities exclusively for COVID-19 patients compared to mixed HCFs, followed by HCFs not dedicated to COVID-19 patients. Tertiary facilities also had better scores followed by secondary and then primary HCFs. While most secondary and tertiary facilities are better equipped in IPC capacity, this study identified gaps in primary HCFs and those not dedicated to COVID-19 patients, whose IPC scores, following assessments, were low. The principal goals of primary HCFs are to ensure quality and safe standards of care that are widespread, comprehensive and accessible to all in the community [14]. However, being the initial contact or first point of care for the community, the primary health care setting is easily exposed to frequent outbreaks, posing a risk to HWs, patients, visitors and the community at large. Treatment centres handling infectious disease outbreaks such as HCFs, designated exclusively for COVID-19 patients are categorized as high-risk facilities in the outbreak settings and hence prioritized for the improvement of IPC capacity at the expense of facilities not handling outbreaks. During the COVID-19 pandemic, there was overt community transmission, with most cases being asymptomatic, causing many HWs to be exposed and disproportionately affected by COVID-19, compared to the general public [6, 15]. Infections among HWs were high in primary HCFs and those not designated for COVID-19 patients, due to inadequate accessibility to IPC enablers such as PPEs, poor risk perception and reduced vigilance or high index of suspicion in these settings [6]. The study highlights gaps and the need to address IPC capacity to respond to COVID-19, health care-associated infections, including AMR across the health system, primary health care settings and non-COVID-19 treatment centres.

HCF that had IPC focal points, IPC guidelines and HWs trained in IPC scored higher than those that did not. In addition, there was a strong correlation between IPC capacity and the presence of IPC focal point in HCFs, the availability of IPC guidelines in HCFs and HCFs that have all its HW trained in basic IPC. The multi-modal strategy highlights the added value of efficiently combining multiple modalities for a synergized response in improving IPC capacity [16, 17]. In this study, the components on IPC focal points, guidelines and training each had an incremental effect in IPC scores, while the combination of the three further raised the scores with an exponential improvement in facilities implementing the three components.

Surveillance of healthcare-associated infections particularly for HW infection is a critical part of IPC. When results of HW infections are timeously reported to managers, there is more likelihood that outbreaks will be detected timeously and mitigation measures established to curb further spread of infection. Some of the HCFs (35.71%) were reported to be experiencing HW risk exposure while others were not. This indicates gaps in HW surveillance and implementation strategies for the protection of HWs in Africa. The health care workforce is the cornerstone of the health system, and is critical to the provision of health care and ensuring continuity of essential quality health care services. Without them, no services are rendered or sustainable even when other parts of the health system are improved [13].

The results in this study are similar to the recently published global IPC survey where the overall Infection Prevention Control Assessment Framework (IPCAF) HCF scores were significantly low in African Region compared to other regions, with the lowest scores in IPC training and education, PPE availability and accessibility [13]. Complimentary to this study and global IPC survey [13], the lack of or limited availability of PPE was also noted in two pulse surveys by WHO on continuity of essential health services the during COVID-19 pandemic [18, 19]. Regarding IPC capacity at different levels of HCFs, the 2019 IPC global survey observed that IPCAF scores in tertiary facilities were higher than in primary HCFs [13].

This is the first study assessing the IPC components in healthcare settings during the COVID-19 pandemic, underscoring the need for other complimentary studies for generalizability and comprehensiveness in reviewing IPC capacity in the African Region. Additionally, the study only looks into the IPC capacity at the facility level. It is paramount to further review capacities at the national level.

## Conclusion

The COVID-19 pandemic was a game changer for IPC, as it highlighted the critical role the latter played in controlling the spread of emerging and re-emerging infections and its relevance in strengthening and building resilience across the health system. Although the study allows us to have an idea of the IPC capacity in HCFs in the African Region, in-depth studies are needed within the Region for generalizability, comprehensiveness and a better understanding of the IPC capacity and areas of support. Despite the global stimulus of the COVID-19 pandemic and its ripple effects, major gaps still exist in IPC in the African Region, especially in primary health care settings and facilities for non-COVID-19 patients. These gaps are likely to hamper the quality and safety of care across the health system. The study highlights defects and the need to improve IPC programmes through strategic and operational interventions aligned to WHO’s minimum IPC requirements.

### Supplementary Information

**Additional file 1: Supplementary material 1. **Infection prevention and control assessment tool for health care facilities in the context of the COVID-19 pandemic.

## Data Availability

Data existed in health facilities and are publicly available. The following link gives access to data used for this article: https://app.powerbi.com/view?r=eyJrIjoiNzVjZWNmMDktZDJmYS00ZjM2LTkzZDgtZTk2NjFlMmQzMDBlIiwidCI6ImY2MTBjMGI3LWJkMjQtNGIzOS04MTBiLTNkYzI4MGFmYjU5MCIsImMiOjh9

## Abbreviations

IPC: Infection prevention and control
HW: Health worker
HCFs: Health care facilities
