# Supplementary material for: Analysing the implementation of infection prevention and control measures in health care facilities during the COVID-19 pandemic in the African Region
Source: BMC Infect Dis. 2023 Nov 23;23:824. doi: 10.1186/s12879-023-08830-8 (PMC10668477; doi:10.1186/s12879-023-08830-8)
Supplement: Supplementary file 1 — Additional file 1: Supplementary material 1. Infection prevention and control assessment tool for health care facilities in the context of the COVID-19 pandemic. [file 12879_2023_8830_MOESM1_ESM.docx]

**Supplementary material 1 :** Infection prevention and control assessment tool for health care facilities in the context of the COVID-19 pandemic

| **ASSESSMENT/THEMATIC CRITERIA** |
| --- |
| **1. IPC programme at the facility** |
| The facility has an identified person, or an IPC focal point, or a Hygiene/IPC Committee who are responsible, accountable, have established terms of reference, known authority, and dedicated time to implement IPC tasks |
| The facility has IPC guidelines according to its service delivery system |
| The facility produces a weekly report of IPC activities implemented |
| **2. Triage available** |
| Temperature and symptoms of the disease are correctly verified - functional ThermoFlash |
| Triage forms, registers, and educational materials (signs, symptom posters) are available and properly utilized and displayed |
| For each open entry point into the health care facility, a triage station is established and operational |
| **3. Identification of an isolation/waiting area** |
| The "isolation" area is easily identified, well ventilated, and separate from other units/services |
| Dedicated latrines/toilets (or individual bedpans/urinals) are available |
| The isolation area contains: hand hygiene facilities, equipment (PPE, bedpan/urinal, etc.), an area for putting on PPE and another for taking off PPE (well respected circuit) |
| **4. Hand washing/Hand washing facilities** |
| Every entrance into the facility, every toilet, and every individual care area contains: hand hygiene station with clean water + soap and/or hydro-alcoholic solution and/or 0.05% chlorinated water solution [when the first two are not available] |
| Staff are able to perform hand hygiene according to WHO guidelines |
| Posters on proper hand hygiene techniques are present at each hand hygiene station |
| **5. Availability and use of personal protective Equipment (PPE)** |
| All PPE are accessible to staff at all times and in sufficient quantities (gloves, masks, aprons/gowns, eye protection, etc., according to the disease requirement ) |
| Posters (standard precautions and disease) on how to put on and take off PPE are visibly displayed |
| Staff are able to put on and take off PPE according to the WHO guidelines |
| **6. Waste segregation** |
| Sealed, covered and labelled (infectious or non-infectious) waste bins and waste management forms are available at all patient service points), |
| Sharps containers are available at all points of use |
| Waste is sorted according to the type of waste (e.g. indicated by colours or labelling): Infectious, non-infectious, sharp (from source, during collection, to disposal and/or treatment) |
| **7. Waste disposal** |
| Staff wear appropriate PPE (gloves, masks, aprons/gowns, eye protection) when handling, separating, and/or transporting waste |
| Waste is burned on site in an incinerator or other treatment system (autoclave), or there is a system for transporting it safely to another suitable location |
| A placenta or organic waste pit is present and used when required |
| **8. Staff training** |
| All staff have been trained on at least standard precautions, additional precautions (practical and theoretical periods) and a focus on the disease within the last 6 months |
| A register is kept containing the names of the health workers who were trained, the training date, the training type, and the organization that provided the training |
| The health worker receives continuing training through on-site supervision |
| **9. Intra-hospital suspicious case alert (at health care facility level)** |
| A hotline is established and well promoted |
| Inpatients are screened at least twice a day to identify suspect cases |
| Once identified, suspect cases are moved to the isolation/transit area and an alert is triggered |
| **10. Sterilization** |
| Sterilized equipment is available (such as autoclave, poupinel and accessories needed for sterilization, PPE) |
| SOPs are available on how to perform sterilization of materials/equipment |
| All health staff performing any sterilization process have been trained |
| **11. Bio-cleaning of the patient's environment** |
| SOPs are available on how to perform cleaning/disinfection (including cleaning schedule and monitoring) |
| Full time health staff performing cleaning and disinfection have been trained (cleaning, disinfecting, HCF waste management) |
| Cleaners wear appropriate PPE according to the disease requirement (gloves, masks, aprons/gowns, eye protection) |
| **12. Health worker exposure or infection with the disease** |
| An assessment and management protocol is in place (including a register, assessment tools, communication) for exposed or confirmed health workers |
| The management of exposed and confirmed health personnel is clearly defined and ensured |
| When a health worker is exposed (or confirmed), an established team is in place, alerted, and begins to perform an investigation |
| **13. Water supply and storage in the health care facility** |
| A functional water supply within the health care facility compound/premises, including purified water point safe for drinking, and water for other usages - handwashing, cleaning, toilets, is available and operational |
| A stand-by storage tank installed in the health care facility to cover water needs for at least 48 hours during main water shortages is available |
| A functional wastewater treatment system onsite (for example, septic tank |
| with outlet to a drainage pit) or wastewater is collected, treated and disposed of offsite |
| **14. Sanitation and hygiene in the health care facility** |
| At least one functional and clean toilet within the health care facility is available |
| Dedicated toilets/latrines for each gender are visible, identifiable and available |
| At least one separate and dedicated toilet for healthcare workers is available (ideally two, sex-segregated). |
